# Gas, Larvae, Coagulopathy, and a Rare Culprit: Emphysematous Cystitis and Enterococcus raffinosus Ventriculitis Triggered by Strongyloides Hyperinfection Syndrome

**DOI:** 10.7759/cureus.82417

**Published:** 2025-04-17

**Authors:** Joshua F Bowyer, David Prentice

**Affiliations:** 1 Internal Medicine, St. John of God Midland Public Hospital, Perth, AUS; 2 Neurosciences, Perron Institute for Neurological and Translational Science, Perth, AUS

**Keywords:** csf infection, empagliflozin, emphysematous cystitis (ec), enterococcus raffinosus, pyogenic ventriculitis, sglt2-inhibitors, strongyloides hyperinfection syndrome, strongyloides stercoralis (ss)

## Abstract

We describe the first documented case of *Enterococcus raffinosus* *(E. raffinosus)* ventriculitis complicating *Strongyloides stercoralis* (SS) hyperinfection syndrome (SHS). A 40-year-old immunosuppressed Indigenous man from a hyperendemic region presented with septic shock from a resistant *Escherichia coli *(*E. coli*) emphysematous cystitis (EC). Diagnostic challenges related to cirrhosis and atypical SS presentation delayed recognition, culminating in ventriculitis confirmed on magnetic resonance imaging (MRI) and cerebrospinal fluid (CSF) culture. Targeted parenteral antibiotics and ivermectin led to full recovery after a prolonged admission and rehabilitation process. This case underscores several critical clinical lessons: empirical daily ivermectin should be strongly considered for patients with sepsis from hyperendemic regions, as it may be lifesaving. Awareness of the hyperendemicity of SS in certain regions of Australia is crucial, as it can precipitate life-threatening septicaemia and central nervous system infections. The cumulative impact of immunosuppressive factors such as diabetes, alcohol dependence, and malnutrition warrants careful evaluation in such patients, as they are easy to overlook. Finally, this case expands our understanding of the pathogenic potential of *E. raffinosus*, a bacterium rarely represented in the current literature.

## Introduction

We report a case of *Strongyloides *hyperinfection syndrome (SHS) complicated by pyogenic ventriculitis caused by a rare pathogen, *Enterococcus raffinosus* (*E. raffinosus*). The patient initially presented with emphysematous cystitis due to extended-spectrum-beta-lactamase (ESBL)-producing *Escherichia coli* (*E. coli*) complicated by septic shock.

SHS is a severe complication of *Strongyloides stercoralis *(*S. stercoralis*) infection characterized by larval dissemination with or without bacterial co-translocation of enteric organisms. This condition can lead to bloodstream infections and dissemination to distant sites, including the lungs and central nervous system (CNS). Whilst SHS is typically associated with significant immunosuppression, it can also occur in individuals with chronic malnutrition, diabetes, and cirrhosis [1-3]. Diagnosing *S. stercoralis *and SHS is challenging, as up to 65% of cases may be seronegative, and many individuals remain asymptomatic [4]. Therefore, in immunocompromised individuals from endemic regions, a high degree of clinical suspicion is crucial [5]. This, in turn, requires an understanding of the epidemiology of *S. stercoralis *infections in Australia [2].

CNS infections in SHS have been reported to involve enteric organisms such as *E. coli*, *Proteus mirabilis*, *Klebsiella pneumoniae*, *Streptococcus bovis*, or *Enterococcus faecalis* [3]. CNS infections caused by "other enterococci" (OE) are rare. In a comprehensive retrospective analysis of enterococcal infections conducted at the Regional Institute of Gastroenterology and Hepatology in Europe, OE accounted for only 17% of all cases (58 out of 339). Among these, only two cases involved the CNS, both presenting as meningoencephalitis, and both were *Enterococcus avium* [6]. *E. raffinosus *is a member of OE and has not been reported as a CNS pathogen at the time of writing based on extensive searches of MEDLINE and PubMed [1,6,7]. Therefore, to the best of our knowledge, this case represents the first documented case of *E. raffinosus *pyogenic ventriculitis.

This report also underscores the diagnostic and therapeutic challenges of managing rare infections in critically ill patients. It also highlights the role common comorbidities such as alcohol misuse and malnutrition may play in triggering SHS and opportunistic infections in patients from endemic regions whilst emphasizing that timely parenteral rather than intraventricular therapy can achieve favourable outcomes in pyogenic ventriculitis despite its often poor prognosis.

## Case presentation

An Indigenous man in his 40s from remote northwestern Australia presented to the local hospital with a three-day history of fever, frequency, and incontinence. His medical history included type 2 diabetes mellitus, a past hepatitis B infection, and a longstanding history of heavy alcohol consumption and tobacco smoking but no intravenous drug use. His prescribed medications were ramipril, atorvastatin, sitagliptin/metformin, and empagliflozin. On examination, he was febrile (38.1°C), hypotensive (85/55 mmHg), and tachycardic (110 bpm).

**Figure 1:**
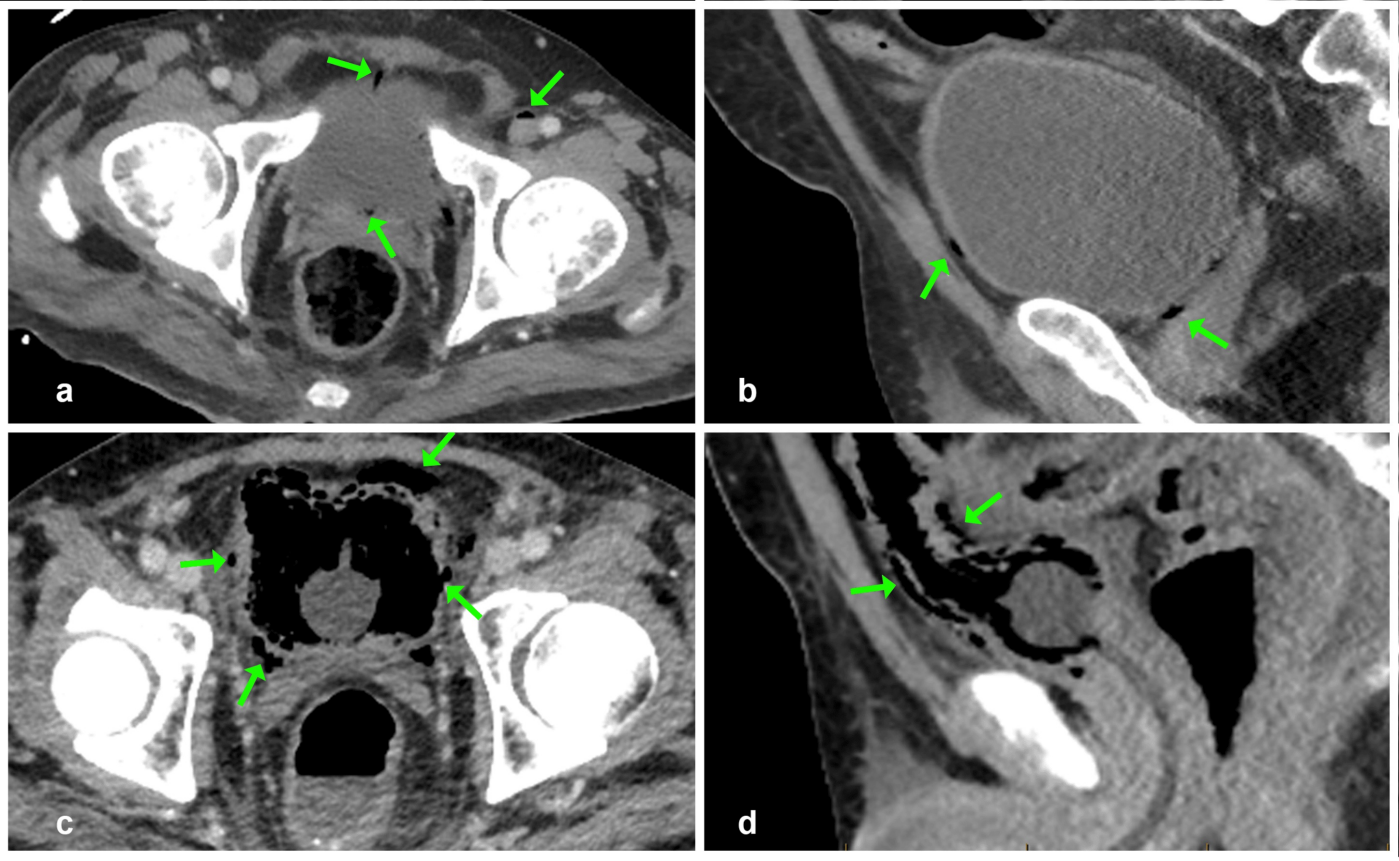
Emphysematous cystitis on CT Initial portal venous phase CT abdomen/pelvis: (a) axial and (b) sagittal views showing linear gas shadow anterior and posterior to the bladder, indicating gas within the pelvic veins. Also, note the presence of gas within the left common femoral vein (green arrows). There is no history of catheterization or attempted line insertion. Figures 1c (axial) and 1d (sagittal) show gas within the bladder lumen and wall, indicating emphysematous cystitis (green arrows).

Initial blood tests revealed a prominent neutrophilia, C-reactive protein elevation, hyperlactatemia, severe hypoalbuminaemia, multiple electrolyte abnormalities, and liver derangements consistent with sepsis-related cholestasis and cirrhotic coagulopathy (Table 1). A portal venous phase CT abdomen/pelvis showed gas within the bladder wall, left common femoral vein, and left internal iliac vein consistent with emphysematous cystitis (Figure 1). Additional findings on CT included ascites and features that were suspected of cirrhosis. A subsequent abdominal ultrasound confirmed cirrhotic changes in the liver, recanalization of the umbilical vein, and ascites, findings consistent with portal hypertension. No other abnormalities were noted on chest X-ray or bedside echocardiography. He was diagnosed with septic shock due to emphysematous cystitis. A urinary catheter was placed to decompress the bladder in consultation with urology. Intravascular volume was restored, and appropriate empirical antibiotics were initiated. Inotropic support was required during air transfer to an intensive care unit (ICU). Initial blood and urine cultures both subsequently grew a multi-resistant ESBL *E. coli*,* *and targeted therapy with intravenous meropenem achieved significant clinical improvement within days.

**Table 1:** Initial blood results ALP: Alkaline phosphatase, GGT: Gamma-glutamyl transferase, AST: Aspartate transaminase, ALT: Alanine transaminase, APTT: Activated partial thromboplastin time, INR: International normalized ratio, HbA1c: Haemoglobin A1c, HBsAg: Hepatitis B surface antigen, HBcT: Hepatitis B core total antibody, HCV: Hepatitis C virus, PCR: Polymerase chain reaction, EBV: Epstein-Barr virus, T. Pallidum: Treponema pallidum

Test	Admission Value	Normal Range
Haemoglobin	97	135-180 g/L
White cell count	37.1	4-11 x 10^9^/L
Neutrophil count	35.6	2–7.5 x 10^9^/L
Lymphocyte count	0.7	1.2-4 x 10^9^/L
Eosinophils	0	<0.7 x 10^9^/L
C-reactive protein	98	<5 mg/L
Lactate	2.5	<1 mmol/L
Lipase	482	29-210 U/L
Calcium	1.74	2.10-2.60 mmol/L
Magnesium	0.52	0.70–1.10 mmol/L
Albumin	13	35-50 g/L
ALP	189	30-110 U/L
Bilirubin	182	<21 μmol/L
GGT	563	5-50 U/L
AST	74	5-35 U/L
ALT	26	5-40 U/L
Sodium	123	135-145 mmol/L
Potassium	3.2	3.5-5.2 mmol/L
Chloride	90	22-32 mmol/L
Bicarb	23	22-32 mmol/L
Creatinine	72	60-110 μmol/L
Prothrombin time	34.5 seconds	9.0-13 seconds
APTT	50.8 seconds	23-35 seconds
APTT 50:50	31.8	50:50 mix normal plasma
INR	2.3	0.8-1.2
Fibrinogen	3.5	2.0-4.0 g/L
Thrombin clotting time	25.8 seconds	<21 seconds
HbA1c	6.5%	<5.7% normal
Respiratory viral PCR	Negative	-
Hepatitis serology (HBsAg, HBcT, HCV)	Not detected	-
Hepatitis serology (HBsAb)	<10 IU/L	HBsAb
EBV antibodies	Negative	IgG and IgM
T. pallidum total antibody	Not detected	-

**Figure 2:**
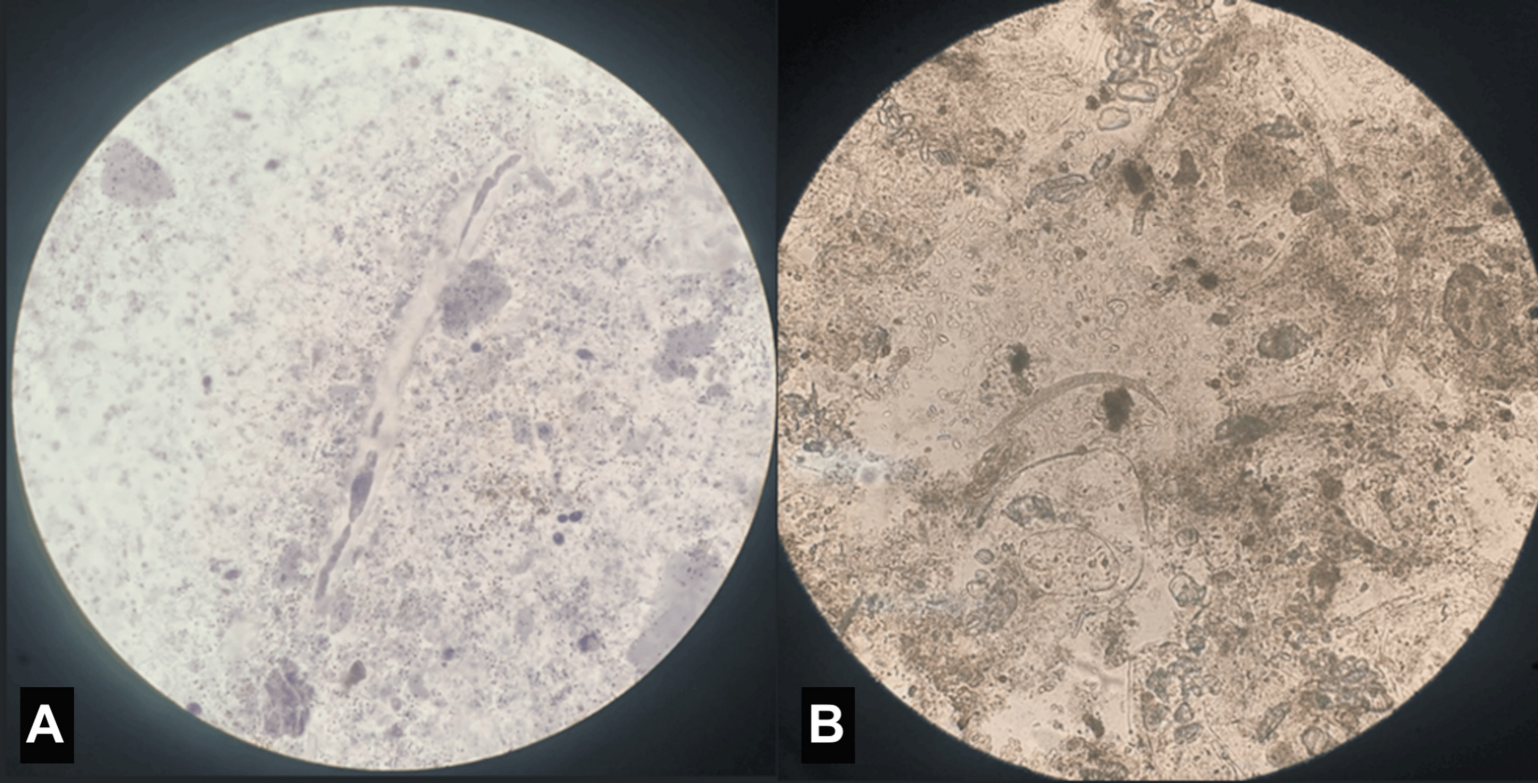
Stool microscopy Strongyloides stercoralis (SS) larvae were detected on stool microscopy at ×40 magnification (A, B).

Over the following weeks, the patient experienced recurrent fevers, hemodynamic instability, and fluctuating consciousness, requiring further ICU-level care. Multiple repeat septic screens were negative. Hepatic encephalopathy and occult collection were suspected, given his newly diagnosed cirrhosis with portal hypertension and mildly elevated ammonia level of 64 µmol/L (normal range: <50 µmol/L), along with favourable responses to aperients and broad-spectrum antibiotics. Further imaging with magnetic resonance imaging (MRI) of the prostate and whole-body fluorodeoxyglucose (FDG) positron emission tomography/computed tomography (PET/CT) were unrevealing. A lumbar puncture was delayed on account of coagulopathy. Although multiple stool specimens were collected from day zero of admission, *S. stercoralis* larvae were detected on only one occasion via stool microscopy on day 25 (Figure 2). This prompted treatment with two doses of ivermectin seven days apart as per Australian therapeutic guideline recommendations [8].

**Figure 3:**
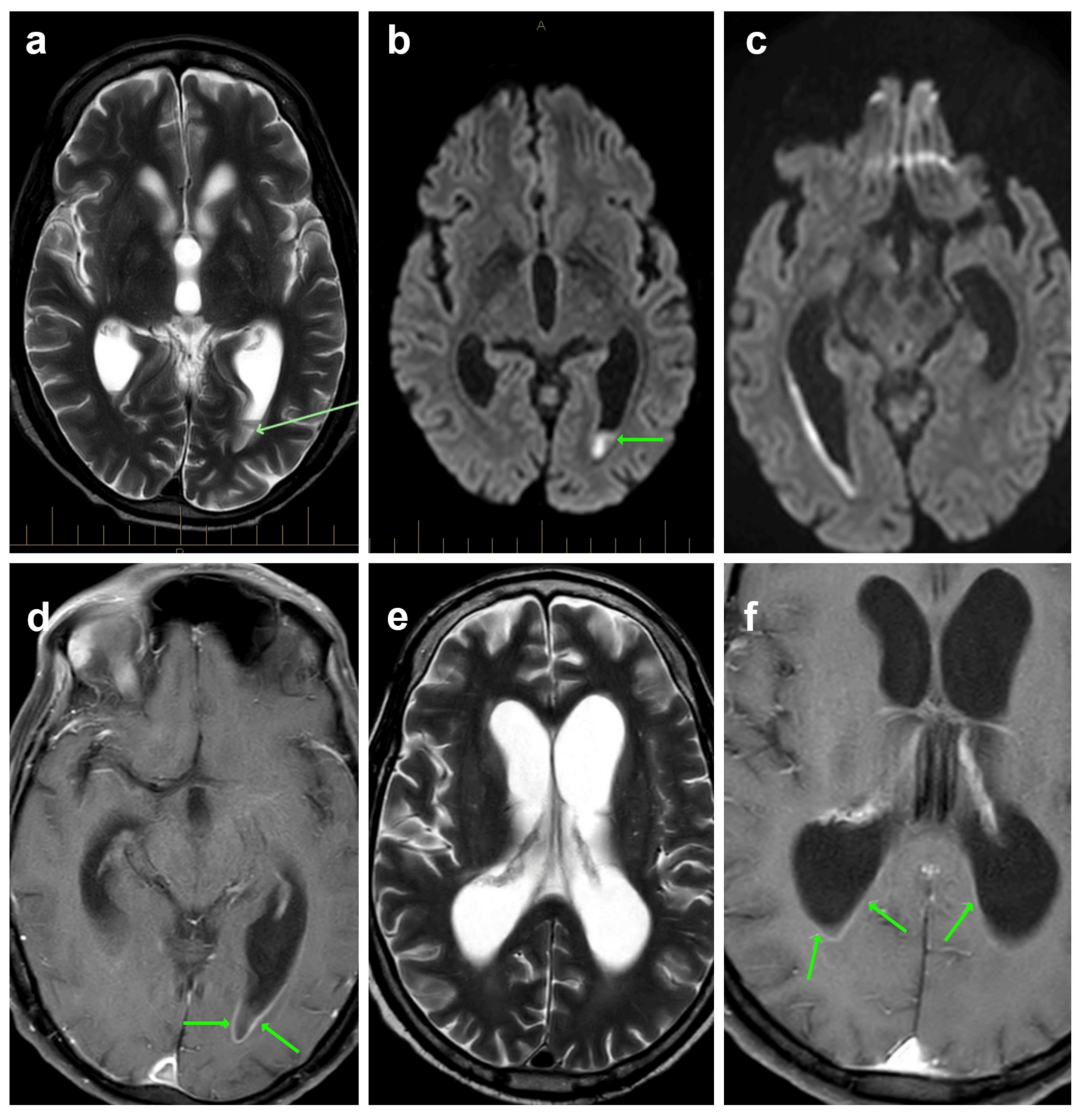
Pyogenic ventriculitis on MRI (a, b) Pus in ventricles in a non-enhanced sequence; (c) diffusion-weighted imaging; (d, f) T1 sequence, post-contrast; (e) T2 sequence demonstrating dilatation of the ventricular system with the smooth ependymal enhancement of the posterior and occipital horns of the lateral ventricles indicating ventriculitis.

After two weeks of gradual clinical improvement, the patient experienced acute-on-chronic neurological deterioration with further decreased consciousness, hemispatial neglect, increased upper limb tone, and reduced lower limb reflexes. An urgent CT brain scan showed early hydrocephalus. Subsequent cranial MRI showed diffusion-restricting fluid-fluid levels in the lateral ventricles, which were initially interpreted as intraventricular haemorrhage. CT angiography showed no vascular abnormality or bleeding point. A follow-up cranial MRI with contrast revealed linear ependymal enhancement in the occipital horns and leptomeningeal enhancement without evidence of bleeding, findings that were consistent with pyogenic ventriculitis (Figure 3). A diagnostic lumbar puncture confirmed bacterial meningitis (Table 2). At this stage, meropenem was used for ESBL *E. coli* pyelitis/emphysematous cystitis with linezolid, fluconazole, ciprofloxacin, and ivermectin for SHS due to concurrent vancomycin-resistant *Enterococcus* (VRE) and candiduria. *E*. *raffinosus* was cultured from the cerebrospinal fluid (CSF) resistant to penicillin and ampicillin but sensitive to vancomycin and ciprofloxacin (Vanc MIC <16 on Vitek, CLSI (Clinical and Laboratory Standards Institute)). Bacterial DNA was confirmed by 16S PCR/NAAT. CSF analysis by wet mount was negative for parasites or filariform larvae. He tested negative for HIV and HTLV-1/2.

**Table 2:** CSF findings pre- and post-treatment An initial lumbar puncture was performed on day 49. Post-treatment lumbar puncture was performed on day 91 following six weeks of therapy. PCR: Polymerase chain reaction, CSF: Cerebrospinal fluid

Parameter	Day 49	Day 91	Reference Range
Protein	1.91	0.84	0.15-0.45 g/L
Glucose	2	3	2.8-4.2 mmol/L
Leucocytes	2490	19	×10^6^/L
Lymphocytes	0	94	(%)
Neutrophils	100%	6%	(%)
Gram stain	Numerous leucocytes, no bacteria	Scanty leucocytes, no bacteria	-
Culture	Moderate growth Enterococcus raffinosus, resistant to penicillin, ampicillin and amoxicillin, sensitive to vancomycin and ciprofloxacin	No growth	-
PCR	Bacterial 16S identification Enterococcus raffinosus	-	-

**Figure 4:**
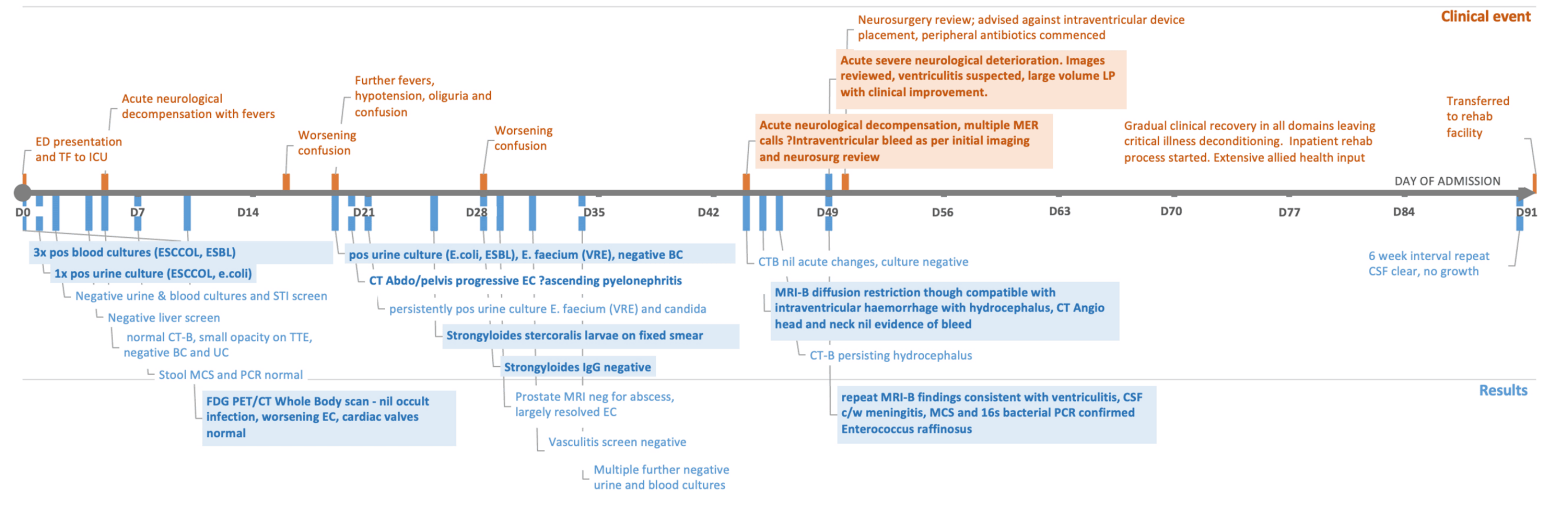
Clinical course of our patient Clinical course from day zero (D0) through day 91 (D91). Timeline of events from admission until transfer to the rehab facility. Relevant clinical events are outlined above the line. Key results are outlined below the line. ESCCOL: E. coli, ESBL: Extended-spectrum beta-lactamase, STI: Sexually transmitted infection, CT-B: Computed tomography brain, TTE: Transthoracic echocardiogram, BC: Blood culture/s, UC: Urine culture/s, MCS: Microscopy, culture, and sensitivity, PCR: Polymerase chain reaction, FDG PET-CT: Fluorodeoxyglucose (FDG) positron emission tomography-computed tomography (PET-CT), pos: Positive, EC: Emphysematous cystitis, VRE: Vancomycin-resistant Enterococcus, E. faecium: Enterococcus faecium, CSF: Cerebrospinal fluid, c/w: "consistent with"

Intraventricular delivery of antibiotics was explored with neurosurgery but deemed too high-risk due to the patient's ongoing coagulopathy. The consensus was for prolonged parenteral therapy with CNS-dosed vancomycin, meropenem, and daily ivermectin in line with therapeutic guidelines [8]. This resulted in steady clinical improvement. He was discharged to rehabilitation whilst completing his course, and interval CSF analysis at six weeks showed biochemical resolution with negative cultures. He returned home approximately three months later without neurological sequelae. His tortuous clinical course is summarized in Figure 4.

## Discussion

We diagnosed our patient with SHS based on multifactorial immunosuppression, polymicrobial sepsis, the presence of *S. stercoralis *larvae in faecal samples, a prolonged and complicated clinical course with recurrent septic flares despite extended therapies, and enterococcal cerebral ventriculitis. Emphysematous cystitis, *S. stercoralis, *SHS and *E. raffinosus *cerebral ventriculitis are discussed.

Emphysematous cystitis

Emphysematous cystitis is a rare complication of urinary tract infections characterized by gas locules within the bladder wall and lumen caused by gas-forming organisms such as *E. coli *through glucose metabolism to carbon dioxide. These pockets are visible on a CT scan, which is the most sensitive test for diagnosis [9]. Mortality ranges from 3% to 12% [10], and it is most often associated with diabetes, alcoholism, and glycosuria (which provides the substrate), all of which were present in our patient. Logically, conditions that amplify glycosuria may exacerbate the problem, and recent reports have linked sodium-glucose transport protein 2 (SGLT2) inhibitors, including empagliflozin, to the development of emphysematous cystitis. Our patient was prescribed empagliflozin [11,12]. Whilst causality remains speculative, SGLT2 inhibitors should be discontinued in patients presenting with emphysematous cystitis, and further research is warranted in this area.

The optimal treatment includes parenteral antibiotics and catheterization to relieve anatomical or functional urinary obstruction, which plays a role in the pathogenesis. In our patient, the presence of gas in the femoral vein was unexpected and raised concern. However, Yoshimatsu et al. reported gas in bladder-draining veins (including iliac vein branches and the femoral vein) in 15 out of 23 patients with *Enterococcus*, with no observed impact on sepsis severity or outcomes. They postulated that intravesical pressurization contributed to this phenomenon [13].

In our patient, the presence of portal hypertension may have contributed to the delay in gas absorption from the bladder wall. Furthermore, it is possible that the invasion of *S. stercoralis* in the mesenteric venous system directly seeded the bladder with bacteria.

Strongyloidiasis, SHS, and diagnostic challenges

In humans, *S. stercoralis* is the most clinically significant species of parasitic nematode from the genus *Strongyloides*. It is endemic in tropical and subtropical regions and is estimated to have infected up to 370 million people worldwide [14,15]. In remote Northern Australian Indigenous populations, it is considered hyperendemic, with seroprevalence reaching up to 60% [4]. Its unique and complex parasitic life cycle means that chronic, asymptomatic strongyloidiasis can persist for decades. In such cases, bouts of immunosuppression, regardless of the cause, can trigger SHS or disseminated infection.

SHS is a severe form of acute strongyloidiasis that occurs in the context of immune suppression. It is characterized by accelerated autoinfection and migration of *Strongyloides *larvae through the gastrointestinal tract and pulmonary system, leading to exacerbation of gastrointestinal and respiratory symptoms. When larvae migrate to other organs such as the CNS, liver, or kidneys, they become disseminated and are more likely to be fatal [4]. Co-carriage of enteric bacteria can result in secondary sepsis and meningitis. Causes of immunosuppression sufficient to trigger such complications include glucocorticoid therapy, organ transplantation, malnutrition, HTLV-1 and HIV infections, alcohol abuse, and cirrhosis [4,16,17]. The latter two are associated with more severe infections and poorer outcomes [14], both of which were present in our patient.

Diagnosing strongyloidiasis and SHS can be difficult. It relies on detecting larvae in stool specimens, but this only has a sensitivity of about 76% on the first assay and is operator-dependent. However, tests with higher sensitivity, such as RT-PCR (reverse transcription polymerase chain reaction) and LIPS (luciferase immunoprecipitation systems) assays, are not widely available. Raised eosinophil counts are not always present, and *S. stercoralis* serology often provides false negatives during an acute infection, as it can take up to six weeks to mount an immune response [17]. There is a widening gap between research in this area and application in clinical practice, which needs to be closed [5].

Eosinophil-dependent mechanisms are involved in killing *Strongyloides *larvae, and the lack of eosinophilia in patients infected with SS is a poor prognostic sign, particularly in immunocompromised patients [18]. In our case, eosinophil levels were initially zero and then showed two small peaks. Initially, it increased to 0.6x10^9^/L on day 25, coinciding with the detection of *S. stercoralis *larvae in stools. Subsequently, a second peak at 1.25x10^9^/L occurred on day 75. The serology test for* S. stercoralis* and multiple stool samples were negative.

The preferred treatment for uncomplicated acute strongyloidiasis is ivermectin 200 µg/kg for two days total. For SHS and disseminated strongyloidiasis, it is 200 mg/kg/day until a negative stool exam persists for two weeks [17]. Our patient was initially treated for uncomplicated acute strongyloidiasis, but his sepsis relapsed. Following the administration of a higher dose of ivermectin, a sustained response to parenteral antibiotics was achieved. In our opinion, this regimen should be considered empirically in similar cases, akin to population-based empirical treatment strategies that have proven effective in endemic regions, where difficulties in diagnosing *S. stercoralis* are common [4].

Pyogenic ventriculitis and therapeutic approach

Ventriculitis is a severe and underreported complication of CNS infections and carries high morbidity and mortality. Prompt diagnosis and aggressive management are essential, as in-hospital mortality approaches 30% and one-year mortality up to 40%. Among survivors, over 60% experience long-term neurological sequelae, including cognitive deficits, epilepsy, or paresis. It most commonly occurs in association with brain abscesses, meningitis, intraventricular devices, or, less commonly, haematogenous spread. Key risk factors include neurosurgical procedures, CNS infections, trauma, immunosuppression, intracranial implants, and hydrocephalus. Community-acquired cases are typically linked to *Streptococcus* species in brain abscesses, whilst less common healthcare-associated infections are typically caused by Enterobacteriales,* Pseudomonas aeruginosa*, or *Staphylococcus *species, especially in the context of intraventricular device insertion or in neuro-perioperative settings. Common symptoms include fever, headache, altered consciousness, seizures, and focal neurological deficits. Diagnosis relies on MRI findings of ependymal enhancement, intraventricular pus, or hydrocephalus. A CSF analysis shows elevated white cell counts, low glucose, and high protein levels. Normal CSF findings do not reliably exclude infection. Management involves a combination of neurosurgical interventions, such as external ventricular drain (EVD) placement, abscess drainage, or device removal, in addition to antimicrobial therapy. Empirical treatment often includes vancomycin (with higher trough levels of 15-20 µg/mL) and anti-pseudomonal antibiotics. The median duration of antibiotic therapy is 21 days [19-22].

In our case, bacterial co-seeding into the CNS during SHS was the presumed mechanism. Intraventricular device placement for antibiotic delivery was contraindicated due to cirrhotic coagulopathy. Instead, prolonged systemic antibiotics, including vancomycin, meropenem, and linezolid, guided by susceptibility testing, successfully resolved the infection. This underscores the utility of systemic therapy as an alternative to intraventricular delivery of antibiotics in select patients.

Novel *E. raffinosus* ventriculitis

*E. raffinosus* is a gram-positive coccus first described in 1989 and is biochemically similar to *E. avium*. It is a rare member of the OE group and often multi-resistant. Its natural habitat is unknown, though it is considered a potential gut commensal. Initially thought not to be pathogenic in humans, it has recently been implicated in bloodstream infections, vertebral osteomyelitis, cholangitis, infective endocarditis, and a peripancreatic collection. Mostly in severely immunosuppressed states such as high-dose corticosteroids, haematological malignancies, and transplant settings [19-21,23-27]. A large study over 14 years at a tertiary centre in Romania found *E. raffinosus* contributed just three (n=3, 1%) of 339 total enterococcal infections. All OE accounted for 17% (n=58/339), and none were from the CNS [7].

To the best of our knowledge and after a comprehensive search, this is the first reported case of ventriculitis caused by *E. raffinosus*, highlighting its potential as an opportunistic CNS pathogen.

Relevance to this case

This case highlights the complex interplay of host and environmental factors leading to SHS and disseminated *S. stercoralis *infection. Our patient lived in a region hyperendemic for *S. stercoralis*,making chronic strongyloidiasis likely. Immunosuppression from diabetes, malnutrition, and alcohol-related cirrhosis was sufficient to trigger SHS, whilst glycosuria from empagliflozin may have played a role in the development of emphysematous cystitis. Cirrhosis-associated intestinal barrier dysfunction (e.g., reduced motility, permeability, and mucosal immunity) likely contributed to the translocation of *E. coli*, resulting in emphysematous cystitis and septic shock [28]. SHS then enabled haematogenous dissemination of *E. raffinosus* into the CNS, probably via the bloodstream.

## Conclusions

This complex case underscores several important diagnostic and therapeutic considerations, particularly in immunosuppressed patients presenting with sepsis from regions wherein *S. stercoralis *is endemic. In such cases, clinicians should maintain a high index of suspicion for SHS, even in the absence of typical markers such as peripheral eosinophilia, gastrointestinal symptoms, or positive serology. Given the potential for rapid deterioration, early empirical treatment with ivermectin at SHS doses may be a low-risk but lifesaving intervention. Ideally, this approach should be in consultation with infectious disease specialists. Additionally, it highlights how the immunosuppressive effects of type 2 diabetes mellitus, alcohol use disorder, and cirrhosis may be underestimated due to their commonality in clinical practice.

Furthermore, in patients with presumed hepatic encephalopathy and cirrhotic coagulopathy, timely diagnostic lumbar puncture should be carefully considered, as the benefits of early identification of central nervous system infections may outweigh the perceived bleeding risks. In the management of pyogenic ventriculitis, this case demonstrates that systemic antibiotic therapy alone can be effective, avoiding the need for intraventricular administration. Finally, the identification of *E. raffinosus* as a causative pathogen adds to the limited existing literature on its clinical significance, reinforcing the need for awareness of its potential pathogenicity.
